# Heart Failure-Smart Life: a randomized controlled trial of a mobile app for self-management in patients with heart failure

**DOI:** 10.1186/s12872-023-03039-8

**Published:** 2023-01-09

**Authors:** Eui-Young Choi, Jin-Sun Park, Deulle Min, Soohyun Ahn, Jeong-Ah Ahn

**Affiliations:** 1 Division of Cardiology, Gangnam Severance Hospital, Yonsei University College of Medicine, Seoul, Korea; 2 Department of Cardiology, Ajou University School of Medicine, Suwon, Korea; 3 Department of Nursing, College of Medicine, Wonkwang University, Iksan, Korea; 4 Department of Mathematics, Ajou University, Suwon, Korea; 5 College of Nursing and Research Institute of Nursing Science, Ajou University, Worldcup-ro 164, Yeongtong-gu, Suwon, 16499 Republic of Korea

**Keywords:** Heart failure, Mobile applications, Self-management, Telemedicine

## Abstract

**Background:**

It is an important strategy for healthcare providers to support heart failure patients with comprehensive aspects of self-management. A practical alternative to a comprehensive and user-friendly self-management program for heart failure patients is needed. This study aimed to develop a mobile self-management app program for patients with heart failure and to identify the impact of the program.

**Methods:**

We developed a mobile app, called Heart Failure-Smart Life. The app was to provide educational materials using a daily health check-up diary, Q & A, and 1:1 chat, considering individual users’ convenience. An experimental study was employed using a randomized controlled trial to evaluate the effects of the program in patients with heart failure from July 2018 to June 2019. The experimental group (n = 36) participated in using the mobile app that provided feedback on their self-management and allowed monitoring of their daily health status by cardiac nurses for 3 months, and the control group (n = 38) continued to undergo their usual care. The differences in the physical, psychosocial, and behavioral factors between the two groups over time were analyzed using the analysis of covariance.

**Results:**

After 3 months of intervention, significant differences between experimental and control groups were shown in the New York Heart Association functional class (*p* = 0.003) and cardiac diastolic function (*p* = 0.024). The improvements over time in the experimental group tended to be higher than those in the control group in considered variables. However, no changes in psychosocial and behavioral variables were observed between the groups over time.

**Conclusions:**

This study provides evidence that the mobile app program may provide benefits to its users, specifically improvements of symptom and cardiac diastolic function in patients with heart failure. Healthcare providers can effectively and practically guide and support patients with heart failure using comprehensive and convenient self-management tools such as smartphone apps.

## Background

Along with the increase in the global aging population, the prevalence of heart failure has rapidly increased. A recent survey by the National Health and Nutrition Examination in the United States estimated the prevalence of heart failure to be 6.2 million, approximately 2.2% of the adult population, between 2013 and 2016 [1]. Despite the development of advanced diagnostic and treatment technologies, patients with heart failure continue to be affected by the disease’s progression rather than ultimately being cured [2]. It has a high rehospitalization rate of patients with heart failure following discharge, with over 20–30% of patients requiring rehospitalization within 30–60 days [3]. Also, patients with heart failure who need to be admitted to the hospital for decompensation have high mortality rates; one in six patients expire during admission or within 30 days after discharge [4]. Thus, developing a strategy for patients with heart failure that reduces hospitalization through lifetime self-management and the independent monitoring of worsening signs and symptoms at home is necessary [5]. Unfortunately, many patients with heart failure lack or have difficulty monitoring their self-management skills in their daily lives [6].

Patients with heart failure have diverse physical symptoms, such as shortness of breath, fatigue, and dizziness, as well as psychological problems of stress and depression, which together result in declines in patients’ overall quality of life (QoL) [7]. Patients’ self-management of heart failure includes their adherence to various behaviors, including medication schedules, healthy dietary habits, safe and regular exercise routines, and awareness of worsening signs and symptoms [8]. However, most patients with heart failure are frail and usually have difficulties in self-management along with physiological and functional decline, psychological problems, and poor QoL [9]. To date, most programs have been designed for use in hospitals with patients having heart failure and are mainly comprised of face-to-face education and exercise training programs, which present challenges for patients due to the requirements of further in-person visits to hospitals and incur additional individual and social medical expenses.

Mobile health apps using smartphones are being introduced to improve healthcare efficiency and patient outcomes in diverse chronic disease management [10, 11]. According to a recent review of apps designed for patients with heart failure, some have been introduced as prototypes or studied for their feasibility, included only the contents of conventional clichés, such as taking medications or tracking symptoms, and often characterized by a lack of interactivity and lack of authoritative information [12]. Another systematic review reported that the studies using a mobile app for heart failure patients measured various physical, psychosocial, and behavioral outcomes, including health status, QoL and self-management behavior, but the results showed inconsistency and imprecision [13]. Thus, it was recommended that future studies are needed for feasibility and efficacy to enhance health care and patient outcomes in diverse populations for generalization of the research using mobile health technology interventions for patients with heart failure [13, 14].

Therefore, this study aimed to develop a comprehensive self-management mobile app program for patients with heart failure and to evaluate the program’s effects on the patient’s physical, psychosocial, and behavioral outcomes.

## Methods

### Study design

This study was a non-blinded, randomized controlled trial (RCT). Data were collected from July 2018 to June 2019. In both the experimental and control groups, data were collected at baseline and 3-month follow-up.

### Participants and recruitment

Participants were recruited from cardiovascular outpatient clinics at two large tertiary medical centers in Korea. One hundred patients were screened for eligibility with the following criteria. Patients were eligible to participate if they were: (1) diagnosed with heart failure by a cardiologist and regularly visiting the hospital clinic for medical follow-ups; (2) between 20–79 years of age; (3) able to use a smartphone app; (4) able to perform regular physical activity according to the patients’ self-identification and the judgment of their primary cardiologist; and (5) willing to participate in the study. Patients suffering from cognitive disorders or participating in another clinical trial were excluded from participation.

We calculated the sample size adequacy using G*Power 3.1 software [15] and arrived at a required total sample size of 55 according to an α level of 0.05, a conventional medium effect size of 0.15, and a power of β = 0.80 for the F-test [16]. Assuming this, the number of participants for this study was considered appropriate.

### Intervention

Based on the international guidelines for the management of heart failure [17, 18], a draft of the possible content for a mobile app was suggested, which included comprehensive self-management content related to lifestyle improvements, such as exercise, nutrition, smoking cessation, stress and daily life management, medication adherence, daily health check-up, and personalized counseling from healthcare providers. We revised this draft after consultation with two cardiologists, three cardiac nurses, and one nursing professor with cardiovascular clinical expertise.

**Fig. 1:**
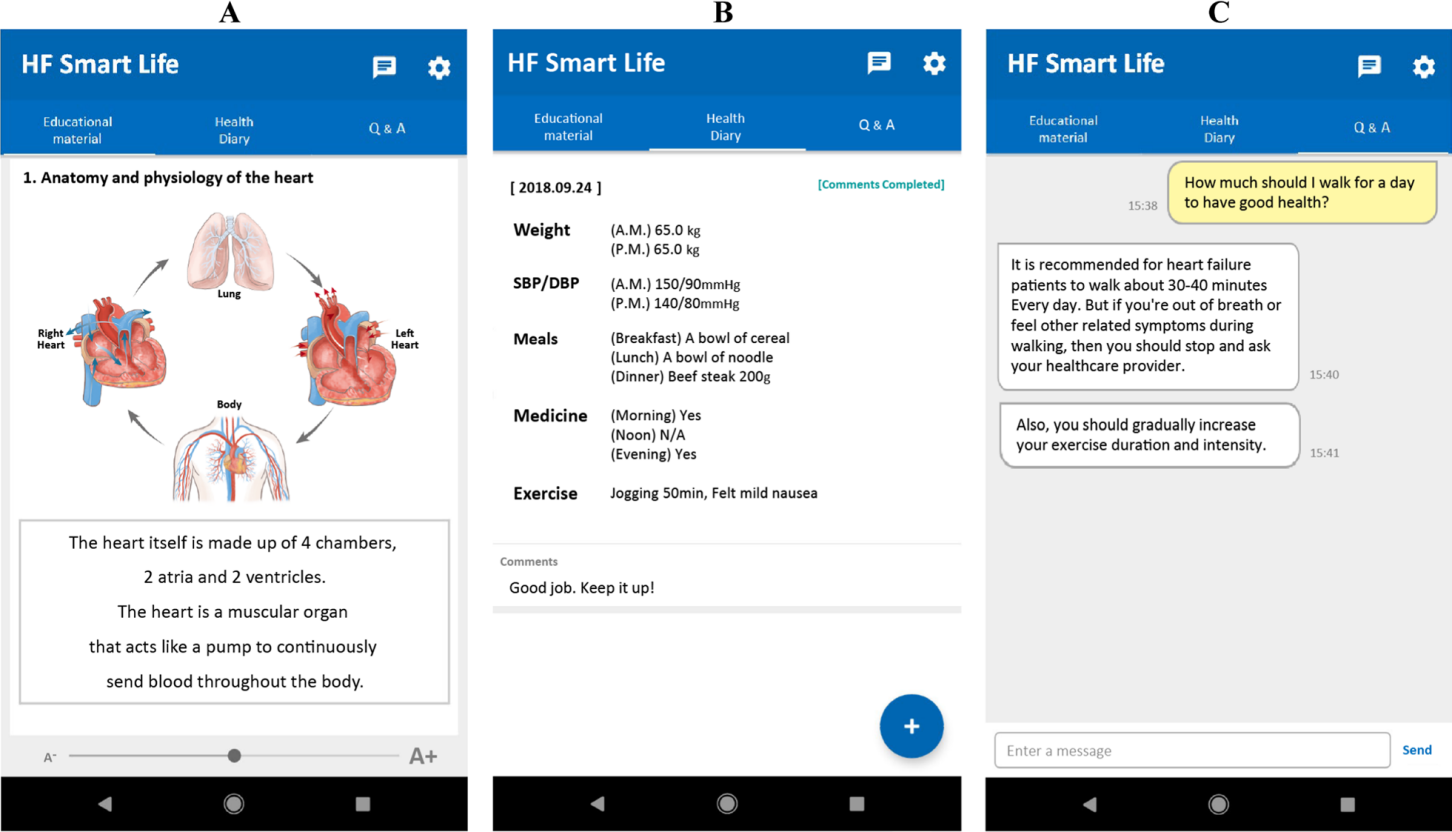
Heart Failure-Smart Life screen features. The app consists of: **A** educational contents; **B** self-management contents with daily health check-ups of weight, blood pressure, nutrition, medication, and exercise; and **C** Q & A and 1:1 chat

The content of the final draft was developed as a mobile app program, named “Heart Failure-Smart Life”, with the cooperation of app development specialists. For comprehensive self-management and efficient communication between heart failure patients and healthcare providers, the Heart Failure-Smart Life installed the configurations and functions of educational materials using intuitive pictures and animations, daily health check-up diary (ie, blood pressure and body weight), Q & A, and 1:1 chat, based on the individual user’s convenience (Fig. 1). In addition, a separate, distinct app and a website for healthcare providers were developed to help overall management of the patients’ app.

In an initial 30-min meeting with the individual patients in the experimental group, a cardiac nurse installed the app of Heart Failure-Smart Life on the patient’s smartphone and explained how to use it appropriately. The nurse encouraged the patients to regularly record their daily health check-up diary, exercise and diet status, medication intake, and any appearance of worsening signs and symptoms on the app. This initial meeting took place in a private room of the cardiovascular outpatient clinic during the patient’s regular hospital visit schedule. After the initial meeting, the same nurse, who performed the patient’s initial meeting, performed regular checks every day for the patient’s record, gave active reminders and feedback on self-management, and communicated with each other via the Q & A and 1:1 chat functions on the app. If newly developed or exacerbated signs and symptoms appeared, an immediate outpatient or emergency room visit was recommended. It was continued for 3 months guided by three cardiac nurses who were trained according to the study protocol prior to the trial.

Those in the control group were explained to continue their usual daily activities and were provided their usual care at the cardiovascular outpatient clinic. It consisted of meeting their cardiologist and cardiac nurse and receiving brief information about medications and the course of their illness, which remained unchanged during the study duration.

### Study variables

We collected baseline demographic and clinical information, including age, gender, the existence of a partner, education level, economic status, duration of heart failure, number of hospital admissions, and comorbidities.

Physiological factors were measured on a three-part form, which included: (1) anthropometric measurements (ie, body mass index [BMI], waist circumference [WC], systolic blood pressure [SBP] and diastolic blood pressure [DBP]); (2) evaluation of the New York Heart Association (NYHA) functional classes I–IV; and (3) transthoracic echocardiography data (ie, left ventricular ejection fraction [LVEF] for LV systolic function and early mitral inflow velocity/tissue Doppler-derived early diastolic mitral annular velocity [E/Ea ratio] for LV diastolic function).

A two-part form recorded psychosocial factors, including (1) depression measured using the Geriatric Depression Scale [19] that included a 15-item questionnaire and (2) QoL measured using the 40-item MacNew Heart Disease Health-Related QoL [20] that included three subscales (ie, emotional, physical, and social QoL).

Two behavioral factors were recorded, which included: (1) medication adherence measured using the 8-item Hill Bone Medication Adherence Scale [21] and (2) self-management behavior measured using the 12-item European Heart Failure Self-Care Behavior [22].

All of the above data were collected by a researcher at baseline as well as 3-month follow-up for all participants during their regular hospital visit schedule.

### Statistical analyses

Data collected in this study were analyzed using SPSS version 23.0 (IBM Corporation, Armonk, NY, USA). It was analyzed by a two-sided test at a significance level of 0.05. The demographic and clinical characteristics of the participants were represented by frequency, mean, and standard deviation. The differences in general characteristics, both demographic and clinical variables, as well as physical (ie, anthropometric measurement, the NYHA functional class, and echocardiographic data), psychosocial (ie, depression and QoL), and behavioral factors (ie, medication adherence and self-management behavior) between experimental and control groups were analyzed using chi-square and independent t-tests. Also, differences in the physical, psychosocial, and behavioral factors between experimental and control groups over the study period were analyzed using analysis of covariance (ANCOVA).

## Results

### Characteristics of participants

**Fig. 2:**
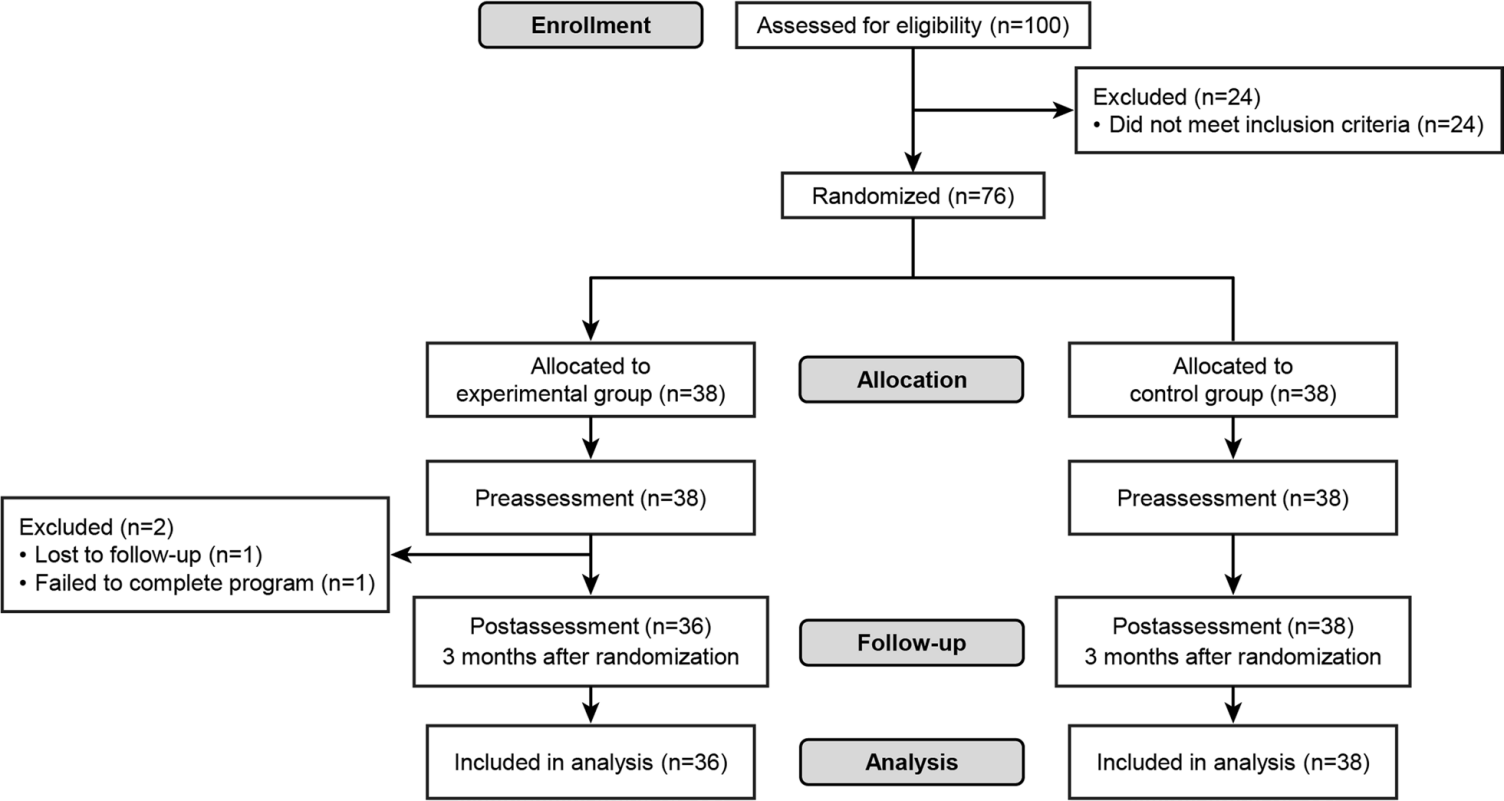
Study flow

We initially screened for eligibility in 100 patients, of which 24 did not meet the inclusion criteria, and 76 patients remained. The remaining 76 patients were randomly assigned to either a control or experimental group by a researcher, with a 1:1 ratio using a computer randomization system [23]. Of the 38 patients assigned to each group, one patient in the experimental group was considered to have failed to complete the program as he did not use the mobile app at least once a week, and the other one in the experimental group was lost to follow-up, while all participants in the control group completed the follow-up. Therefore, the final sample was 36 and 38 patients in the experimental and control groups, respectively (Fig. 2).

Baseline demographic and clinical characteristics are presented in Table 1. There was a difference in the mean age between the two groups (experimental group: M = 70.31 years, SD = 10.55; control group: M = 79.42 years, SD = 7.59; *p* < 0.001). Other than age, there were no differences in other demographic and clinical characteristics between the two groups.

**Table 1:** Homogeneity of demographic and clinical characteristics between groups (*N* = 74)

Characteristics	Experimental group (*n* = 36)	Control group (*n* = 38)	*p*
Age (y), *M* (*SD*)	70.31 (10.55)	79.42 (7.59)	< 0 .001
Gender, *n* (%)			
Male	19 (52.8)	16 (42.1)	0.493
Female	17 (47.2)	22 (57.9)	
Spouse, *n* (%)			
Yes	26 (72.2)	24 (63.2)	0.559
No	10 (27.8)	14 (36.8)	
Education level, *n* (%)			
≤ Middle school	18 (50.0)	26 (68.4)	0.245
High school	12 (33.3)	7 (18.4)	
≥ College	6 (16.7)	5 (13.2)	
Economic status, *n* (%)			
Low	8 (22.2)	10 (26.3)	0.610
Middle	19 (52.8)	22 (57.9)	
High	9 (25.0)	6 (15.8)	
Duration of heart failure (y), *M* (*SD*)	7.92 (5.37)	7.41 (5.33)	0.684
Number of admissions, *M* (*SD*)	0.83 (0.81)	1.26 (2.04)	0.234
Hypertension*, *n* (%)			
Yes	26 (74.3)	35 (92.1)	0.083
No	9 (25.7)	3 (7.9)	
Diabetes*, *n* (%)			
Yes	9 (25.7)	14 (36.8)	0.441
No	26 (74.3)	24 (63.2)	

*Excluded, no response

Baseline outcome variables are presented in Table 2. Anthropometric measurements (BMI, WC, and DBP) did not significantly differ between the two groups, except for SBP at baseline (experimental group: M = 126.19 mmHg, SD = 13.92; control group: M = 133.79 mmHg, SD = 14.66; *p* = 0.025). There were no significant differences in the NYHA functional class, echocardiographic data, depression, QoL, medication adherence, and self-management behavior between the two groups at baseline.

**Table 2:** Homogeneity of outcome variables between groups at baseline

Variables	Experimental group (n = 36)	Control group (n = 38)	*p*
*Physiological factors*			
Anthropometric measures			
Body mass index (kg/m^2^), *M* (*SD*)	25.14 (3.78)	24.65 (4.11)	0.591
Waist circumference (cm), *M* (*SD*)	89.25 (8.52)	88.03 (11.62)	0.609
Systolic blood pressure (mmHg), *M* (*SD*)	126.19 (13.92)	133.79 (14.66)	0.025
Diastolic blood pressure (mmHg), *M* (*SD*)	77.56 (10.72)	75.26 (11.79)	0.385
NYHA functional class, n (%)			
I	6 (16.7)	2 (5.3)	0.051
II	21 (58.3)	15 (39.5)	
III	7 (19.4)	15 (39.5)	
IV	2 (5.6)	6 (15.8)	
Echocardiographic data			
LVEF (%), *M* (*SD*)	64.50 (10.01)	66.00 (7.57)	0.497
E/Ea ratio, *M* (*SD*)	12.24 (4.87)	14.70 (4.87)	0.055
*Psychosocial factors*			
Depression, *M* (*SD*)	4.97 (4.10)	5.45 (3.51)	0.593
Quality of life, *M* (*SD*)	5.62 (8.16)	5.45 (0.86)	0.381
*Behavioral factors*			
Medication adherence, *M* (*SD*)	8.61 (1.42)	8.26 (0.69)	0.189
Self-management behavior,* M* (*SD*)	41.17 (6.85)	44.16 (7.47)	0.077

*NYHA* the New York Heart Association; *LVEF* left ventricular ejection fraction; *E/Ea* early mitral inflow velocity/early diastolic mitral annular velocity

### Effects of Heart Failure-Smart Life mobile app program

Of the 36 participants in the experimental group, 24 (66.7%) reported that they had participated in the mobile app program regularly (more than 5 days per week), including the participation in recording health check-up diary, 1:1 chat with the cardiac nurse, physical activity and exercise, dietary change, medication intake, and monitoring signs and symptoms according to the guidelines on the app by the 3 months follow-up. The average number of usage was 45.79 times over 3 months.

Table 3 shows the effects of the Heart Failure-Smart Life mobile app program using ANCOVA to control for baseline age and SBP. The evaluation of the patients’ physiological factors indicated that significant differences in the NYHA functional class (*p* = 0.003) and LV diastolic function (E/Ea ratio) (*p* = 0.024) existed between the two groups. In addition, the NYHA functional class was significantly improved over time (*p* = 0.040). While the interaction effects of time and group were not statistically significant, the improvement in the NYHA functional class in the experimental group (from 2.14 to 1.82; mean difference = 0.32) was higher than that of the control group (from 2.66 to 2.38; mean difference = 0.28) at the 3 months follow-up. E/Ea ratio for LV diastolic function in the experimental group decreased from 12.24 to 11.35 (mean difference = 0.89) but increased from 14.70 to 16.42 (mean difference = -1.72) in the control group after 3 months. Other physiological variables, which were not significant, included BMI, WC, SBP, DBP, and LVEF.

**Table 3:** Effects of the Heart Failure-Smart Life program

Factors*	Group	Baseline	3-month	Group effect	Time effect	Interaction effect
		M (SD)	M (SD)	*p*	*p*	*p*
*Physiological factors*						
Anthropometric measures						
Body mass index (kg/m^2^)	Exp	25.14 (3.78)	25.74 (3.14)	0.480	0.437	0.929
	Cont	24.65 (4.11)	25.29 (4.14)			
Waist circumference (cm)	Exp	89.25 (8.52)	90.75 (7.42	0.647	0.403	0.871
	Cont	88.03 (11.62)	89.90 (11.88)			
Systolic blood pressure (mmHg)	Exp	126.19 (13.92)	126.93 (14.10)	0.050	0.624	0.464
	Cont	133.79 (14.66)	130.46 (19.07)			
Diastolic blood pressure (mmHg)	Exp	77.56 (10.72)	77.04 (11.75)	0.057	0.264	0.696
	Cont	75.26 (11.79)	71.58 (13.24)			
NYHA functional class	Exp	2.14 (0.76)	1.82 (0.72)	0.003	0.040	0.986
	Cont	2.66 (0.82)	2.38 (0.98)			
Echocardiographic data						
LVEF (%)	Exp	64.50 (10.01)	64.24 (11.15)	0.421	0.587	0.555
	Cont	66.00 (7.57)	62.93 (9.26)			
E/Ea ratio	Exp	12.24 (4.87)	11.35 (4.12)	0.024	0.659	0.229
	Cont	14.70 (4.87)	16.42 (7.99)			
*Psychosocial factors*						
Depression	Exp	4.97 (4.10)	4.86 (4.10)	0.205	0.617	0.812
	Cont	5.45 (3.51)	5.04 (3.74)			
Quality of life	Exp	5.62 (0.82)	5.62 (0.78)	0.209	0.735	0.771
	Cont	5.45(0.86)	5.34 (1.09)			
*Behavioral factors*						
Medication adherence	Exp	8.61 (1.42)	8.46 (1.00)	0.608	0.686	0.268
	Cont	8.26 (0.69)	8.58 (1.39)			
Self-management behavior	Exp	41.17 (6.85)	48.14 (6.07)	0.478	< 0.001	0.141
	Cont	44.16 (7.47)	47.12 (7.31)			

*Adjusted for baseline age and systolic blood pressure. Exp., experimental group; Cont., control group; NYHA, the New York Heart Association; LVEF, left ventricular ejection fraction; E/Ea, early mitral inflow velocity/early diastolic mitral annular velocity

Regarding psychosocial factors, depression and QoL showed no significant differences between the two groups after 3 months. Regarding behavioral factors, there were no significant differences in medication adherence between the two groups after 3 months. As for self-management behavior, the mean score of the experimental group increased from 41.17 to 48.14 after 3 months, and that of the control group changed from 44.16 to 47.12. There were significant differences over time (*p* < 0.001) but not between the two groups.

## Discussion

This study developed a mobile app of the Heart Failure-Smart Life that is a comprehensive self-management program for patients with heart failure and evaluated the effects of the program through a RCT design. It demonstrated no changes in the primary outcomes of psychosocial (depression and QoL) and behavioral factors (medication adherence and self-management behavior), but there seemed to be beneficial effects on the secondary outcomes of patients’ NYHA functional class and cardiac diastolic function.

Mobile apps have the advantage of providing flexibility in obtaining and using health-related information at a pace desired by the patients, regardless of time and place [24]. Also, mobile apps can be an effective strategy to help individual patients with chronic illnesses by encouraging them to easily and reliably monitor the symptoms, recording the symptoms, and independently review them, allowing patients to actively participate in their own treatment process and enhance their positive collaborations with healthcare providers [25]. However, while there are currently a variety of mobile health apps, few available apps are specifically tailored for patients with heart failure [26]. Further, many existing health apps were not developed by healthcare professionals, so they may not be clinically appropriate, or their reliability may be in question [27]. The mobile app, Heart Failure-Smart Life, presented in this study was developed through the collaboration between health professionals (cardiologists, cardiac nurses, and a nursing professor) who had clinical cardiovascular expertise and experts in app development. It included online educational materials focused on heart failure management that reflect international guidelines. Further, the app utilized individual and customized feedback regarding self-management for patients with heart failure by designated nurses in this study. It helped encourage self-management and the monitoring of patients’ basic health status and worsening signs and symptoms in their daily lives. By incorporating a smartphone, the app functioned as a practical and convenient method for regular health checks and communications between patients and their healthcare providers. Traditionally, the key roles for nurses were providing patients with face-to-face nursing care and health education. A potential function of mobile app for healthcare providers will not be only for traditional patient care but for delivery of individual health education, providing free and repeated access to health information, personal monitoring and recording, and enabling faster communication among healthcare providers and the patients without limitation of geographic distance [28].

In the present study, the participating patients utilized the Heart Failure-Smart Life app and showed improvement tendency in the NYHA functional class and E/Ea ratio. The NYHA classification system grades patients’ functional class from having no restriction of physical activity because of one’s symptoms (Class I) to having symptoms typical of heart failure (Class IV) [29]. The classifications reflect the development or progression of heart failure and are widely used as an important tool for outcome evaluation and prognostic value regarding mortality [30]. Various studies reported improvements in patients’ functional class after single or combination of medical therapy [31, 32]; however, few studies have shown significant outcomes related to the functional class through non-pharmacological and short-term interventions, such as the present study. In a recent systematic review of mobile-based interventions in patients with heart failure [33], one study reported that a home-based telemonitoring intervention was significantly improved the patients’ NYHA class [34]. Establishing evidence regarding the beneficial effects of non-pharmacological interventions, especially mobile-based trials, on patients’ functional class is needed.

Furthermore, the present study found that the patients who participated in the Heart Failure-Smart Life app showed a significant improvement in their E/Ea ratio. E/Ea ratio is the value of LV diastolic function measured by the noninvasive echocardiographic estimation, an essential evaluation tool as a strong prognostic indicator in patients with heart failure [35]. Further, high E/Ea is associated with negative outcomes for patients with heart failure, such as readmission and mortality risk [36]. According to a meta-analysis of eight exercise intervention studies [37], endurance training alone or in combination with strength training was beneficial for both exercise capacity and diastolic function. We emphasized the importance of regular exercise and its impact on cardiac health through online education as a part of the Heart Failure-Smart Life mobile app. Also, the patients performed and recorded their exercise as a self-management behavior, and the nurses provided positive reinforcement for them. This series of processes could have a positive effect on the E/Ea ratio of the participants.

However, the Heart Failure-Smart Life mobile app program did not show any differences between the two groups in a number of the physiological, psychosocial, and behavioral outcomes measured in the present study. There has been controversy regarding results for those outcomes in previous studies, but above all, a possible reason for the present findings is that the intervention duration was only 3 months. This length of time may be insufficient to change the measured psychological and behavioral factors in patients with heart failure.

Although the importance of self-management in the treatment of heart failure is being emphasized generally by healthcare providers, it is not easy for patients to maintain it efficiently. Nevertheless, mobile apps could be a practical strategy for improving and maintaining self-management capabilities for patients with heart failure. In particular, considering the pandemic era, such as the COVID-19 pandemic, the availability of non-face-to-face strategies, like mobile apps, will be critical, and their impact on patients who are remaining at home should be investigated. Through further clinical trials using mobile apps, it can expand the potential to give patients with diverse chronic diseases better engagement and ownership of lifelong self-management at home [38] and enable to meet the expectations of healthcare providers to optimize the patients’ therapeutic adherence in the future.

There are several limitations in this study to note. First, the study was a RCT study with a small sample. Possibly because of this small sample size, significant differences that could affect the prognosis of heart failure were observed in the baseline characteristics, such as age and SBP between the experimental and the control groups. Moreover, the interaction effects of time and group were not detected even some variables showed the different patterns over time between the two groups. Second, we could not collect information about the patient’s medications and laboratory findings, such as serum or urine sodium levels, that may affect the study results. Third, the intervention period of 3 months is a relatively short period for assessing psychosocial and behavioral changes of the patients. Future studies are needed that include a larger number of participants, more extensive patient information collection, and an extended period of intervention and follow-up. In addition, the usability of the mobile app for chronic disease self-management can be further increased through the app development process that reflects the expectations and needs of users and includes prior user testing [39].

## Conclusions

A mobile-based self-management app, the Heart Failure-Smart Life, implied improvements in the experimental group’s functional class and cardiac diastolic function. Mobile apps appear to benefit patients’ continuous self-management and health outcomes, healthcare providers’ consecutive monitoring, effective communication with each other, and further collaboration to achieve better heart failure treatment goals in the future.

## Data Availability

The data that support the findings of this study are available from the corresponding author upon reasonable request and with permission of the medical centers where the authors collected the data retrospectively.

## Abbreviations

ANCOVA: Analyses of covariance
BMI: Body mass index
DBP: Diastolic blood pressure
E/Ea: Early mitral inflow velocity to early diastolic mitral septal annular velocity
LV: Left ventricle
LVEF: Left ventricular ejection fraction
NYHA: The New York Heart Association
QoL: Quality of life
RCT: Randomized controlled trial
SBP: Systolic blood pressure
WC: Waist circumference
